# IMPACT WV: Adapting Patient Navigation Models of Care to Improve Services to Caregivers of Infants Exposed to Substances in Utero

**DOI:** 10.13023/jah.0701.09

**Published:** 2025-05-01

**Authors:** Charlotte S. Workman, Lesley Cottrell, Mawyah Bashatah, Aisha Hashmi, Kayla Richard, Poornima Murthy, Susan Harrison, Ashleigh McKinsey, Amy Bott, Sydney Leary, Cody Smith, Melina Danko

**Affiliations:** WVU Reserach Corp; West Virginia University; West Virginia University; United Hospital Center

**Keywords:** Appalachia, Neonatal Abstinence Syndrome, Patient Navigator Model, Perinatal Substance Use, Research in Rural Settings

## Abstract

Patient navigation (PN) is a healthcare service delivery model designed to partner clients with service providers to reduce challenges to health care, as well as to improve access to care by identifying opportunities to coordinate services for clients. The IMPACT WV project at the West Virginia University Center for Excellence in Disabilities (WVU CED) implemented PN models of care for caregivers of infants exposed to substances in utero. The purpose of this program was to reduce burden for potentially at-risk families and improve caregiver and infant outcomes as a result. To understand the processes that influence the implementation of PN models of care with this population, the project modified existing surveys developed by the Fox Chase Virtual Health Cancer Program.

Patient navigation (PN) is a healthcare service delivery model designed to partner clients with service providers to reduce challenges to health care, as well as to improve access to care by identifying opportunities to coordinate services for clients. The IMPACT WV project at the West Virginia University Center for Excellence in Disabilities (WVU CED) implemented PN models of care for caregivers of infants exposed to substances in utero. The purpose of this program was to reduce burden for potentially at-risk families and improve caregiver and infant outcomes as a result. To understand the processes that influence the implementation of PN models of care with this population, the project modified existing surveys developed by the Fox Chase Virtual Health Cancer Program.

To collect data, surveys were distributed in April 2022 to patient navigators (PNs), families, and providers to assess the effectiveness of the service model. Questions were designed to gather characteristics, satisfaction, months experience working with a PN, and outcomes of the PN services.

## Overall satisfaction with PN service delivery

**Figure 1:**
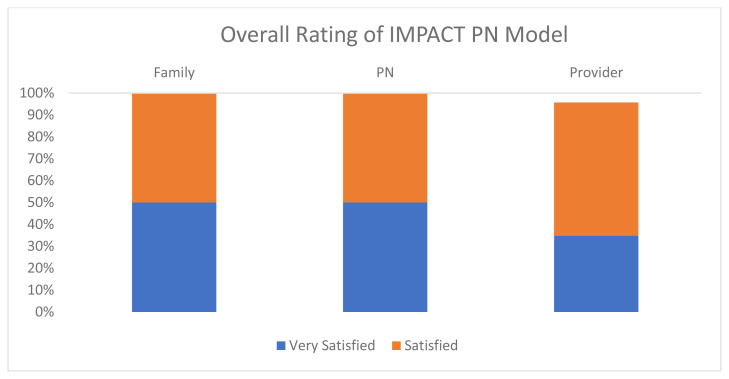
Perceived Satisfaction with PN Model

Perceived satisfaction with the PN model differed by respondent group (Fig. 1). PNs and providers were comfortable with the role; most of the feedback about the patient navigation model was positive, with stakeholders largely being satisfied with the model and activities. While the response rate among families was lower than other partners, families largely felt very satisfied with the services they received, particularly the support coordinating services.

Each respondent group overwhelmingly perceived a strong support for the program (p=.705) and for their patient navigator experience (p=.616). Families utilizing the model largely spoke positively about their interactions with the PN model. Many instances of the navigator “knowing what I needed before I needed it” were documented. Families suggested navigators could strengthen the outreach and enrollment processes a little more, noting they were not always sure where to go if they were interested in services after seeing a flyer or brochure. Additionally, most PNs (three; 75.0%) felt addressing barriers to services was important for a PN to accomplish. Navigators focused on balancing the documentation and service pieces of their position. Their interactions with families were pivotal to their satisfaction with their job. Finally, providers recommended strengthening the program by talking more about it. Specifically, providers felt sharing information about the program to hospital administrators and leaders would lead to greater interest and sustainability of the model within the clinical setting. Similarly, programming referrals and services within electronic medical systems would remind providers while meeting with families who could benefit from a PN.

Qualitative data was coded in NVivo to evaluate the value of the PN. Sixteen overall themes resulted from items evaluated on the family and provider surveys. The value of the PN was noted through guidance (40.07%), helpfulness (98.85%), aid (58.78%), benefit (58.78%), and support (77.50%). Respondents included foster (four; 66.7%), biological (one; 16.67%) and kinship (one; 16.67%) caregivers. Two (33.3%) respondents had been providing care for 0 – 6 months, one (16.6%) for 7–13 months, two (33.3%) for 13 – 18 months, and one (16.67%) for more than 19 months. Four female PNs completed the survey. Three (75%) PNs had provided services ten years or less; one (25.0%) had provided services for more than 21 years. Less than half of providers (9; 28.13%) worked with the model for ten years or fewer; 12 (37.5%) had worked between 21 – 30 years; 6 (18.74%) of respondents had worked 31 years or more.

Initial perceptions of the implementation paralleled the definition of PNs, noting that by enhancing coordination between health care and social services, the model helps to navigate complex systems, working to improve health outcomes.

## Conclusion

Limitations include the small sample size, which suggests the need for future research to be conducted. This future research would ideally expand survey use on a larger scale in an effort to continue identifying the need for implementation alongside outcomes of existing PNPs. Plans also involve looking at the PN model in the context of the Plan of Safe Care for WV. This plan would serve to expand coordination of maternal healthcare services using a healthcare delivery model that aims to overcome individual barriers to care.
